# Four new species of the genus *Lyroda Say* (Hymenoptera, Crabronidae) from China, with a key to Chinese species

**DOI:** 10.3897/zookeys.1279.187068

**Published:** 2026-05-15

**Authors:** Chengfang Li, Qiang Li, Li Ma

**Affiliations:** 1 Department of Entomology, College of Plant Protection, Yunnan Agricultural University, Kunming, Yunnan, 650201, China Department of Entomology, College of Plant Protection, Yunnan Agricultural University Kunming China

**Keywords:** Digger wasp, key, *
Lyroda
*, taxonomy

## Abstract

This paper presents a systematic study on the Chinese species of the genus *Lyroda* Say. Four new species of *Lyroda* from Yunnan, Guangxi, and Zhejiang provinces are described and illustrated: *L. multidentis* Li & Li, *L. retirugosa* Li & Li, *L. curvicarina* Li & Li, and *L. quadratidens* Li & Li. Diagnostic characters distinguishing these species from their congeners are provided. A key to known and new species of the genus *Lyroda* from China is provided.

## Introduction

The genus *Lyroda* (Hymenoptera, Crabronidae, Crabroninae, Miscophini) was first established by Say in 1837, with *Lyroda subita* Say, 1837 as its type species. These wasps are ground nesters and provision their nests with the cricket families Gryllidae (used by *L. subita*) and Tetrigidae (used by *L. japonica* Iwata, 1933, *L. formosa* (Smith, 1858), and *L. madecassa* Arnold, 1945) of the order Orthopyera (Tsuneki and Iida 1969). The method of transporting prey varies: most species grasp prey by the antennae with their mandibles and fly directly or in short glides (Patton 1892; Iwata 1963, 1964), while *L. japonica* is noted for carrying prey with its midlegs (Tsuneki and Iida 1969). *Lyroda subita* may either walk or fly with prey and typically nests in pre-existing burrows or cavities (Evans 1964). Nests are long, deep burrows with the final cell located 15 to 30 cm below the surface, and the entrance is left open during foraging (Evans 1964; Tsuneki and Iida 1969). Nests can contain more than one cell (Evans 1964; Tsuneki and Iida 1969). The egg is laid on the last provision placed in the cell (Tsuneki and Iida 1969), and the developing larvae feed on the cached prey. These wasps are parasitized by miltogrammine flies (Evans 1964).

The genus *Lyroda* currently comprises 26 recognized species and four subspecies, distributed across all six major zoogeographical regions. This genus is primarily distributed in the Oriental region (11 species, two subspecies) (Cameron 1889; Bingham 1905; Williams 1928; Giner Marí 1945; Bibby 1947; Tsuneki 1967; Tsuneki 1983a; Porter et al. 1999; Li and He 2004; Li et al. 2009; Mawadda et al. 2021; Mawadda and Girish Kumar 2023), followed by the Australasian region (four species, one subspecies) (Schulz 1908; Turner 1914; Turner 1916; Turner 1936), the Afrotropical region (three species) (Kohl 1894; Arnold 1945; Schmid-Egger and Al-Jahdhami 2021), the Palearctic region (two species, one subspecies) (Iwata 1933; Tsuneki 1983b; Schmid-Egger 2021), and both the Nearctic and Neotropical regions with two species each (Say 1837; Smith 1856). In addition, one species is distributed in both the Oriental and Palearctic regions, and one species occurs in the Oriental, Palearctic, and Australasian regions (Smith 1858; Williams 1928; Tsuneki 1967, 1983c; Guichard 1980). In China, five species and three subspecies have been previously recorded (Tsuneki 1967; Pu 1986; Wu et al. 2003; Li and He 2004; Li et al. 2009).

In the current study, four new species of *Lyroda* from China are described and illustrated: *L. multidentis* Li & Li, *L. retirugosa* Li & Li, *L. curvicarina* Li & Li, and *L. quadratidens* Li & Li. An identification key to the *Lyroda* species of China is also provided.

## Materials and methods

The specimens examined during this study were collected from Yunnan, Guangxi and Zhejiang provinces in China and deposited in the Insect Collection of Yunnan Agricultural University, Kunming, P. R. China (YNAU). The specimens were observed and illustrated using an Olympus stereomicroscope (SZ Series) with an ocular micrometer. The photographs were taken with a Keyence VHX-5000 digital microscope.

The following abbreviations used in the text are defined as follows:

**A3** Length of antennal segment 3;

**BL** Body length as measured from anterior margin of head to posterior margin of second tergum;

**F** Flagellar articles;

**Gs** Gastral sternum;

**Gt** Gastral tergum;

**HL** Head length;

**HW** Head width;

**IODv** Interocular distance at base of vertex;

**Od** Anteriorocellus distance;

**ODD** Posteriorocellus distance;

**OOD** Ocellocular distance;

**PD** Puncture diameter;

**PIS** Puncture interspaces;

**POD** Postocellar distance, minimum distance between the posterior ocelli.

### Taxonomy

#### Genus Lyroda

Taxon classificationAnimaliaHymenopteraCrabronidae

Say, 1837

2C0120E2-1C89-57D9-B000-B53BB843EF6C

##### Type species.

*Lyroda subita* Say: 1837, subsequently designated by Patton, 1880.

##### Diagnosis.

The main diagnostic characteristics of the genus *Lyroda* include inner orbits of compound eyes parallel, converging medially in upper part in few species; female clypeus with two or more lateral teeth on anterior margin, male clypeus with anterior margin usually truncate or with a median quadrangular tooth; inner margin of mandible with two sub-basal teeth in female, and edentate in male; outer-ventral margin of mandible with notch or angle in both sexes; forewing with three submarginal cells (except *L. errans* (R. Turner, 1936)) with two submarginal cells), second submarginal cell trapezoidal, sessile, bounded by 4–6 veins; marginal cell of forewing truncate apically; pronotum with three tuberculate projections; propodeum elongate, dorsal enclosure delimiting groove inconspicuous, usually with median carina, some species with lateral carinae; fore tarsi of both sexes with conspicuous rake-like structure; gaster sessile (except very rarely with fleshy stem); and both sexes with pygidial plate on apical gastral tergum.

### Key to the species of the genus *Lyroda* from China

Females of *L. curvicarina* Li & Li and *L. quadratidens* Li & Li; males of *L. multidentis* Li & Li, *L. retirugosa* Li & Li and *L. salai* Giner Marí are unknown.

**Table:** 

1	Female (antenna 12 segments; anterior margin of clypeus with two or more lateral teeth)	**2**
–	Male (antenna 13 segments; anterior margin of clypeus without lateral tooth)	**11**
2	At least two gastral segments red (Australasian, Oriental and Palearctic regions)	***L. formosa* (Smith, 1858)**
–	Gaster entirely black (excluding apical margin of terminal segment and pygidium)	**3**
3	Anterior margin of clypeus with three sets of three large teeth (intermediate teeth absent, inconspicuous, or well defined)	**4**
–	Anterior margin of clypeus with two sets of three large teeth laterally, and a set of two large medial teeth (intermediate teeth absent, inconspicuous, or well defined)	**5**
4	Clypeus with one intermediate tooth between large lateral and median tooth sets; length relation between abscissae of radial vein of forewing: 3 < 1 (China: Yunnan)	***L. retirugosa* Li & Li, sp. nov**.
–	Clypeus with two or three intermediate teeth between large lateral and median tooth sets; length relation between abscissae of radial vein of forewing: 3 = 1 (China: Yunnan)	***L. tridentata* Li, Cai & Li, 2009**
5	Clypeus without intermediate teeth between large lateral and median tooth sets	**6**
–	Clypeus with intermediate teeth between lateral and median tooth sets	**7**
6	Clypeal anterior margin slightly projecting medially; Gs 1–2 yellowish-brown; propodeum densely punctate (China: Taiwan)	***L. nigra takasago* Tsuneki, 1967**
–	Clypeal anterior margin with a very weak apical emargination medially; Gs 6 yellowish-brown; propodeum coriaceous (Oriental and Palearctic regions)	***L. nigra japonica* Iwata, 1933**
7	Clypeus with one intermediate tooth between large lateral and median tooth sets	**8**
–	Clypeus with more than one intermediate tooth between large lateral and median tooth sets	**9**
8	Setae on body silvery; basal rounded platform of first tergum not conspicuously concave (Oriental region)	***L. venusta* Bingham, 1897**
–	Setae on frons, pronotum, scutum and propodeum conspicuously brassy; basal rounded platform of first tergum conspicuously concave (China: Fujian, Taiwan)	***L. venusta taiwana* Tsuneki, 1967**
9	Median set of clypeal teeth wide, not cuspidate; forewing without blackish fascia crossing two submarginal cells (Oriental region)	***L. williamsi* Tsuneki, 1983**
–	Median set of clypeal teeth narrow and cuspidate; blackish fascia crossing two submarginal cells and apex of second discoidal cell in forewing	**10**
10	Setae on clypeus, frons, pronotum, scutum and propodeum golden, dense (China: Yunnan)	***L. multidentis* Li & Li, sp. nov**.
–	Setae silvery, dense or sparse (Oriental region)	***L. salai* Giner, 1945**
11	Base of gaster at least I–II red (Australasian, Oriental and Palearctic regions)	***L. formosa* (Smith, 1858)**
–	Gaster entirely black (excluding apical margin of terminal segment and pygidium)	**12**
12	Propodeum without median longitudinal carina, or with a very weak median carina not reaching apex, apex without transverse carina (China: Taiwan)	***L. nigra takasago* Tsuneki, 1967**
–	Propodeum with conspicuous median longitudinal carina, apex with transverse carina	**13**
13	Propodeum with oblique wrinkles or ridges or transverse wrinkles on each side of median carina (Oriental and Palearctic regions)	***L. nigra japonica* Iwata, 1933**
–	Propodeum with reticulate wrinkles or ridges on each side of median carina	**14**
14	Anterior margin of clypeus gently excavated; median tooth less developed (Oriental region)	***L. williamsi* Tsuneki, 1983**
–	Anterior margin of clypeus distinctly, arcuately excavated; median tooth well developed	**15**
15	Sternum VIII without median tooth or protuberance at apex; pygidial plate truncate apically	**16**
–	Sternum VIII with median tooth at apex; pygidial plate rounded apically	**18**
16	Clypeal anterior margin teeth medially slightly concave; Gs2 with a pair of round ferruginous maculae (China: Yunnan)	***L. tridentata* Li, Cai & Li, 2009**
–	Clypeal anterior margin teeth without medial concavity; Gs2 without round ferruginous maculae	**17**
17	Vertex sculptured; fourth abscissa of radial vein of forewing equal to first abscissa; discontinuous silvery band of pile on terga 1–3 (China: Yunnan)	***L. curvicarina* Li & Li, sp. nov**.
–	Vertex not sculptured; fourth abscissa of radial vein of forewing greater than first abscissa; continuous silvery band of pile on terga 1–3 (China: Guangxi)	***L. quadratidens* Li & Li, sp. nov**.
18	Pygidial plate not incised at apex, its lateral carina somewhat rounded (Oriental region)	***L. venusta* Bingham, 1897**
–	Pygidial plate incised at apex, its lateral carina not rounded (China: Fujian, Taiwan)	***L. venusta taiwana* Tsuneki, 1967**

#### Lyroda multidentis

Taxon classificationAnimaliaHymenopteraCrabronidae

Li & Li sp. nov.

4EF5AE46-4E63-5CAA-87EA-9151D0388EFB

https://zoobank.org/6769DF02-3B20-4CE5-B126-9CAFF9E3265F

Fig. 1

##### Type material.

***Holotype*. China** • ♀; Yunnan, Xishuangbanna Tropical Botanical Garden, Rubber Plantation; 21°55'N, 101°15'E; 31.V–3.VII.2019; 550 m elev.; coll. Ling Zhao; Malaise trap (YNAU). ***Paratypes***. • 2♀♀; same location as holotype, 18.VII–20.VIII.2018 (1♀); 14.VII–14.VIII.2018 (1♀); **China** • 4♀♀; Yunnan, Jinghong City, Menghai County, Guanggang Village, Farmland 1; 21°49'N, 100°29'E; 24.VIII–23.IX.2019 (2♀♀); 15.VIII–15.IX.2020 (2♀♀); 1580 m elev.; coll. Ling Zhao; Malaise trap (YNAU); **China** • 2♀♀; Yunnan, Jinghong City, Menghai County, Guanggang Village, Farmland 2; 21°49'N, 100°29'E; 15.VIII–15.IX.2019; 1580 m elev.; coll. Ling Zhao; Malaise trap (YNAU); **China** • ♀; Yunnan, Xishuangbanna Tropical Botanical Garden, Rainforest 2; 21°53'N, 101°12'E; 14.VII–14.VIII.2020; 794 m elev.; coll. Ling Zhao; Malaise trap (YNAU).

**Figure 1.**
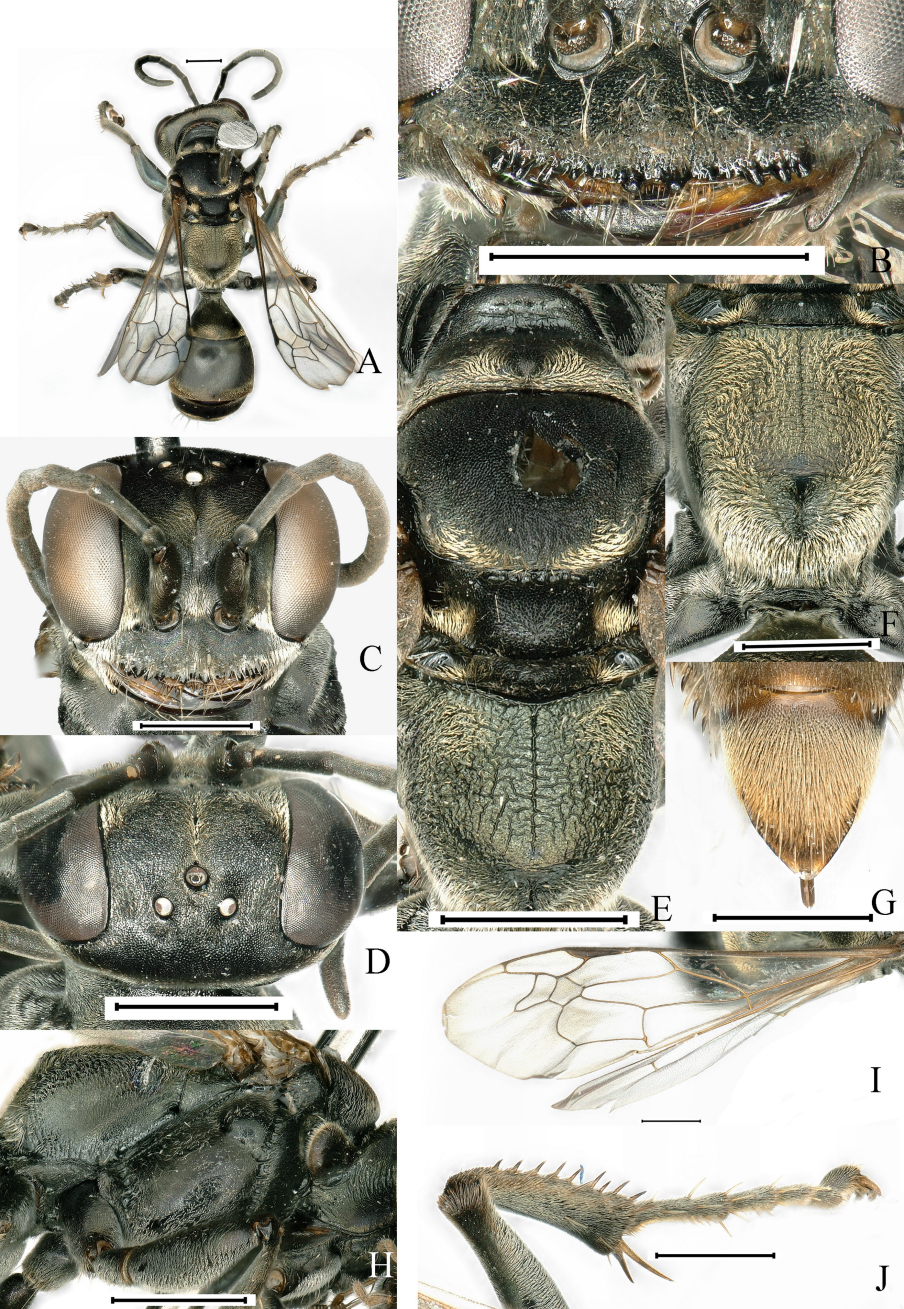
*Lyroda multidentis* Li & Li, sp. nov. Female. Holotype. **A**. Habitus, dorsal view; **B**. Clypeus and mandible; **C**. Head, frontal view; **D**. Head, dorsal view; **E**. Pronotum, scutum and scutellum, dorsal view; **F**. Propodeum, posterodorsal view; **G**. Pygidial plate, dorsal view; **H**. Thorax, lateral view; **I**. Forewing; **J**. Hind leg. Scale bars: 1.0 mm.

##### Diagnosis.

This new species is similar to *Lyroda nuda* Mawadda & Girish Kumar, 2021, but can be distinguished by the following characteristics: in the new species, anterior margin of the clypeus with two blunt teeth and two groups of two median teeth each; the second flagellomere longer than the scape; gastral terga 1–3 with bands of golden setae; and the lateral areas of the propodeum with coarse and discontinuous oblique rugae. To the contrary, in *L. nuda* anterior margin of clypeus with two sharp teeth and two groups of three median teeth each; the second flagellomere equal in length to the scape; gastral terga 1–3 with bands of silvery-white setae; and the lateral areas of propodeum shagreened and punctate.

##### Description.

**Female. *Measurements***. ♀, BL 10.2–12.0 mm. HW: HL: IODv: A3 = 21: 16: 11: 6; ODD: POD: Od: OOD = 10: 26: 12: 27.

***Color pattern***. Black; base of mandible, tibial spurs and tarsal claws more or less ferruginous; wings hyaline, slightly infumated at two external submarginal cells and at apex of second discoidal cell, veins testaceous, stigma blackish brown. Setae golden; dense on clypeus margin, gena, frons, vertex behind eye, tibiae, femora, posterior and posterolateral area of scutum, lateral margins of scutellum and metanotum, all sides of propodeum, and sternal areas of thorax; discontinuous golden band of pile on Gt2–Gt3, setae extensively distributed on Gt1, more densely distributed at apex, forming band of pile; pygidium with dense, stiff brown setae mixed with longer erect brown bristles.

***Head***. Base of mandible deeply cut near base to give rise to two teeth-like prominences, distinctly incised at base on outer margin. Clypeus sparsely punctate (PIS > PD), coriaceous; anterior margin of clypeus with two subobtuse teeth medially and two sets of three prominent teeth at lateral margins, with two sets of three intermediate teeth (Fig. 1B). Frons densely, finely punctate (PIS < PD), clearly divided into two parts by median frontal sulcus. Vertex densely covered with small punctures (PIS > PD) (Fig. 1D). Inner orbits of eyes parallel at vertex. Antenna thin, F2 longer than scape.

***Mesosoma***. Pronotal collar lower than scutum, with three prominent tubercles (Fig. 1E), propleuron with oblique furrow containing conspicuous carinae (Fig. 1H); scutum with fine, dense punctures, PIS on average less than PD, anterior third of scutum with two medial parallel carinae diverging posteriorly; scutum with medial longitudinal depression; scutellum and metanotum with fine, dense punctures, PIS on average more than PD (Fig. 1E). Mesopleuron convex, sparsely covered with shallow punctures (PIS > PD) (Fig. 1H). Propodeal dorsum with median carina extending close to posterior margin without short transverse carina, lateral to median carina with reticulate carinae (Fig. 1E), posterior surface with narrow wedge-shaped deep median furrow, sides of furrow with somewhat sinuate rugae (Fig. 1F); lateral surface of propodeum with rough and discontinuous oblique rugae (Fig. 1H).

***Legs***. Hind tibia with 7–9 spines on posterior external margin (Fig. 1J).

***Wings***. Forewing with infumation crossing two submarginal cells and apex of the second discoidal cell; length relation between abscissae of radial vein of forewing: 2 ≤ 5 < 3 < 1 < 4 (Fig. 1I).

***Metasoma***. Basal platform of first segment approximately obconical with constriction, having conspicuous edges; gaster micropunctate with microscopic setae, surface alutaceous and shiny; pygidial plate with lateral carina (Fig. 1G).

**Male**. Unknown.

##### Distribution.

China (Yunnan).

##### Etymology.

The specific epithet *multidentis* is derived from the Latin “*multi*-” (many) and “*dentis*” (tooth), meaning “many-toothed” and referring to the multi-toothed on the anterior margin of clypeus of this species.

#### Lyroda retirugosa

Taxon classificationAnimaliaHymenopteraCrabronidae

Li & Li sp. nov.

608F6A0A-B834-588F-B767-543F29707962

https://zoobank.org/DE222802-51D7-4DDA-9584-7C4916F1634C

Fig. 2

##### Type material.

***Holotype*. China** • ♀; Yunnan, Jinghong City, Menghai County, Guanggang Village, Farmland 1; 21°49'N, 100°29'E; 24.VIII–23.IX.2019; 1580 m elev.; coll. Ling Zhao; Malaise trap (YNAU). ***Paratypes*. China** • ♀; Yunnan, Xishuangbanna Tropical Botanical Garden, Orchard; 21°53'N, 101°12'E; 24.VII.2016; 794 m elev.; coll. Ling Zhao; Yellow Pan Trap (YNAU).

##### Diagnosis.

This new species is similar to *Lyroda binghami* Tsuneki, 1983 but can be distinguished by the following characteristics: in the new species, anterior clypeal margin with a single small tooth on each side between the lateral and median tooth groups; frons glabrous; lateral area of propodeum shagreened and shiny, spiracular groove conspicuous with a coarse carina above it. To the contrary, in *L. binghami*, anterior margin with 2–3 small teeth on each side between the lateral and median tooth groups; frons densely pubescent, lateral area of propodeum densely punctate with dull interstices (though overall surface shiny), spiracular groove inconspicuous and without carina above it.

##### Description.

**Female. *Measurements***. ♀, BL 11.0–12.1 mm. HW: HL: IODv: A3 = 26: 19: 14: 6; ODD: POD: Od: OOD = 5: 18: 7: 9.

***Color pattern***. Black; mandible, tibial spurs and tarsal claws more or less ferruginous; wings hyaline, veins testaceous, stigma, blackish brown. Setae silvery; dense on anterior margin of clypeus, gena, tibiae, femora, posterior and posterolateral area of scutum, lateral margins of scutellum and metanotum, all sides of propodeum, and sternal areas of thorax; discontinuous silvery band of pile on Gt2–Gt3, setae extensively distributed on Gt1, more densely distributed at apex, forming band of pile; pygidium with dense, stiff brown setae mixed with longer erect brown bristles.

**Figure 2.**
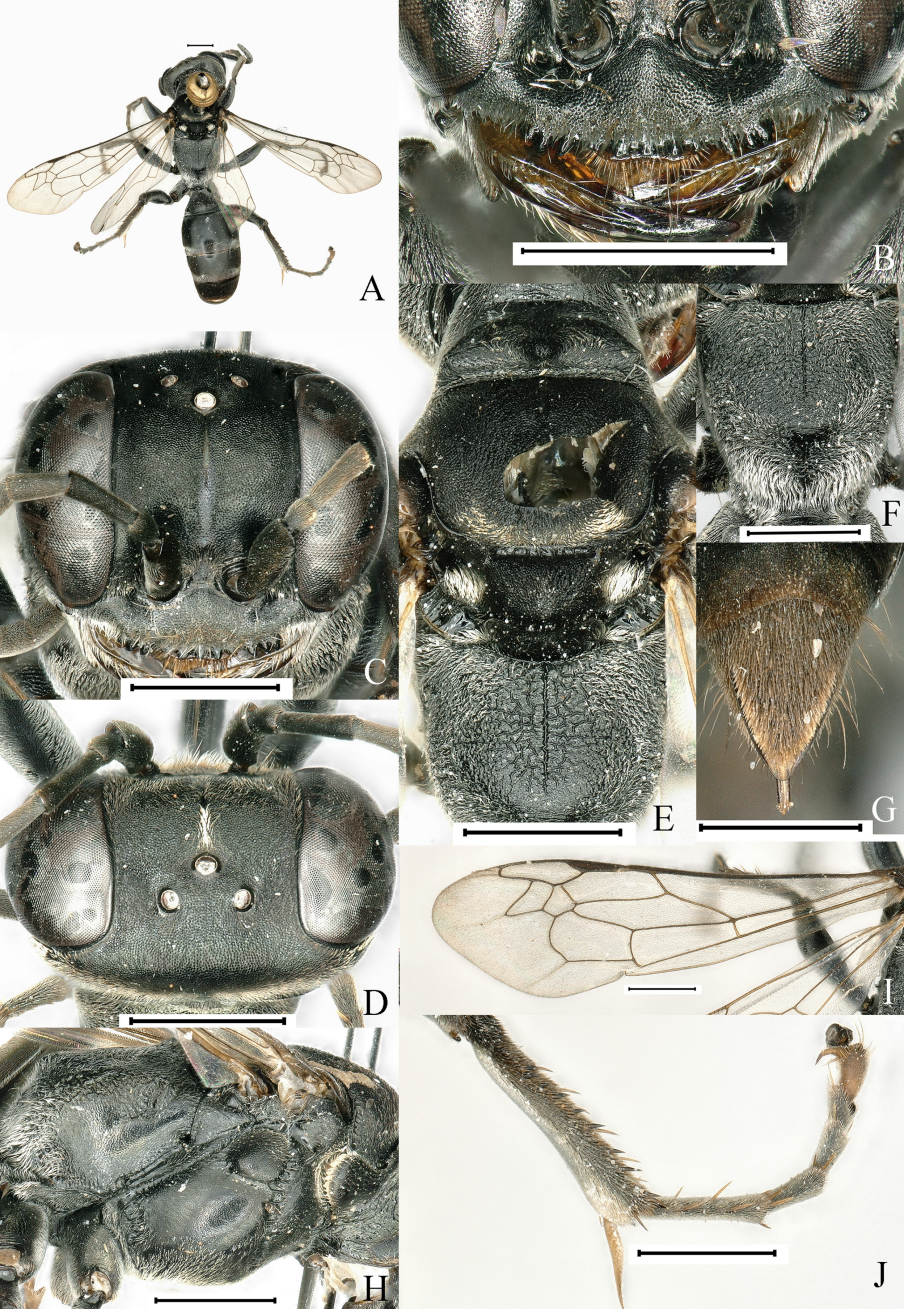
*Lyroda retirugosa* Li & Li, sp. nov. Female. Holotype. **A**. Habitus, dorsal view; **B**. Clypeus and mandible. **C**. Head, frontal view; **D**. Head, dorsal view; **E**. Pronotum, scutum and scutellum, dorsal view; **F**. Propodeum, posterodorsal view; **G**. Pygidial plate, dorsal view; **H**. Thorax, lateral view; **I**. Forewing; **J**. Hind leg. Scale bars: 1.0 mm.

***Head***. Base of mandible deeply cut near base to give rise to two teeth-like prominences, distinctly incised at base on outer margin (Fig. 2B). Clypeus densely, finely punctate (PIS > PD); anterior margin of clypeus with three cuspidate teeth medially and two sets of three prominent teeth at lateral margins, with two sets of one intermediate teeth (Fig. 2B). Frons densely, finely punctate (PIS < PD), clearly divided into two parts by median frontal sulcus. Vertex densely covered with small punctures (PIS > PD), coriaceous (Fig. 2D). Inner orbits of eyes parallel at vertex. Antenna thin, F2 longer than scape.

***Mesosoma***. Pronotal collar lower than scutum, with three prominent tubercles (Fig. 2E), propleuron with oblique furrow containing conspicuous carinae (Fig. 2H); scutum with fine dense punctures, PIS on average less than PD, anterior one third of scutum with two medial parallel carinae diverging posteriorly; scutum with medial longitudinal depression; scutellum and metanotum densely, finely punctures, PIS on average more than PD (Fig. 2E). Mesopleuron convex, with sparse, shallow punctures (PIS > PD) (Fig. 2E). Propodeal dorsum with median carina extending close to posterior margin, posterior margin without conspicuous short transverse carina; lateral to median carina with reticulate carinae (Fig. 2E), posterior surface with narrow wedge-shaped deep median furrow, sides of furrow with somewhat sinuate rugae (Fig. 2F). Upper part of the lateral area of the propodeum with irregular rugae, and the remainder finely punctate and the interspaces shiny (Fig. 2H).

***Legs***. Hind tibia with 7–9 spines on posterior external margin (Fig. 2J).

***Wings***. Forewing without infumation crossing two submarginal cells and apex of the second discoidal cell; length relation between abscissae of radial vein of forewing: 2 ≤ 5 < 3 < 1 < 4 (Fig. 2I).

***Metasoma***. Basal platform of first segment approximately obconical with constriction, having conspicuous edges; gaster micropunctate with microscopic setae, surface alutaceous and shiny; pygidial plate with lateral carina (Fig. 2G).

**Male**. Unknown.

##### Distribution.

China (Yunnan).

##### Etymology.

The specific epithet *retirugosa* is derived from the Latin “*reti*-” (net) and “*rugosa*” (wrinkled), meaning “net-wrinkled” and referring to the propodeum of this species, which has a reticulate rugosity.

#### Lyroda curvicarina

Taxon classificationAnimaliaHymenopteraCrabronidae

Li & Li sp. nov.

C03677BB-E128-5E8F-8607-EC4840A8AB79

https://zoobank.org/C72970A9-5F17-4E80-89CD-06F69B42334F

Fig. 3

##### Type material.

***Holotype*. China** • ♂; Yunnan, Jinghong, Nabang River Basin National Nature Reserve; 22°09'N, 100°39'E; 19–23.VI.2011; 720 m elev.; coll. NaSen Wei; Yellow Pan Trap (YNAU). ***Paratypes***. • 6♂♂; same data as for holotype.

**Diagnosis**. This new species is similar to *L. tridentata* Li, Cai & Li, 2009 can be distinguished by the following characteristics: in the new species, median tooth of the clypeal anterior margin without a notch and the lateral margin of clypeus forming an obtuse angle, both Gt2 and Gs2 without circular ferruginous spots; Gt13 with bands of golden setae, and the fourth abscissa of radial vein equal to or longer than the first. To the contrary, in *L. tridentata* median tooth of the clypeal anterior margin with a small notch and lateral margin forming an acute angle, Gt2 and Gs2 with a pair of ferruginous spots, gastral tergites 1–3 with bands of silvery-white setae, and the fourth abscissa of radial vein shorter than the first.

##### Description.

**Male. *Measurements***. ♂, BL 9.1–10.0 mm. HW: HL: IODv: A3 = 20: 18: 13: 5; ODD: POD: Od: OOD = 3: 9: 4: 8.

***Color pattern***. Black; mandible, tibial spurs and tarsal claws more or less ferruginous; wings hyaline, slightly infumated at two external submarginal cells and at apex of second discoidal cell, veins testaceous, stigma blackish brown. Setae golden; dense on clypeus, base of mandible, antenna, gena, frons, vertex behind eye, tibiae, femora, posterior and posterolateral area of scutum, lateral margins of scutellum and metanotum, all sides of propodeum, and sternal areas of thorax; discontinuous golden band of pile on Gt2–Gt3, setae extensively distributed on Gt1, more densely distributed at apex, forming band of pile; pygidium with dense, stiff brown setae mixed with longer erect brown bristles.

**Figure 3.**
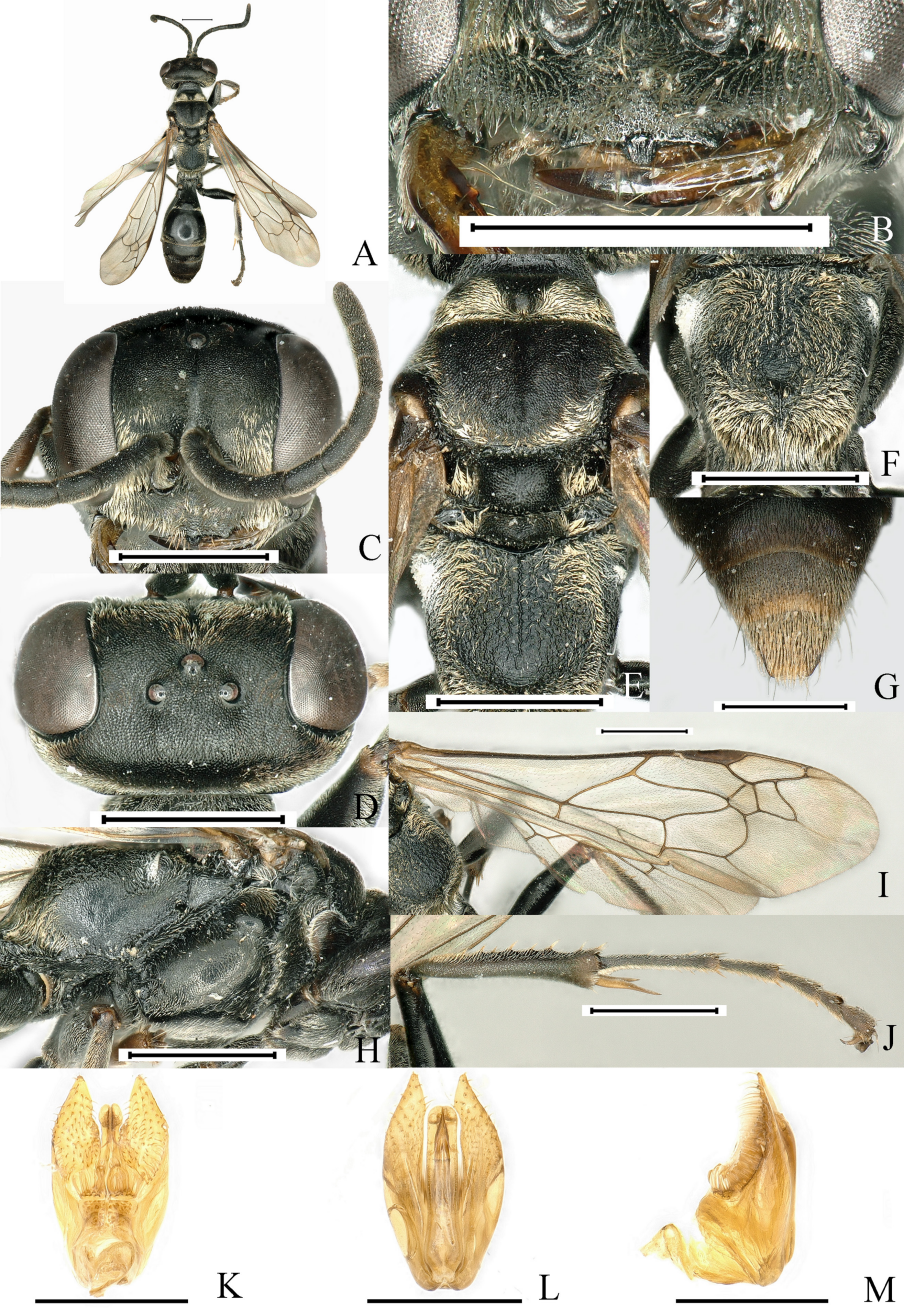
*Lyroda curvicarina* Li & Li, sp. nov. Male. Holotype. **A**. Habitus, dorsal view; **B**. Clypeus and mandible; **C**. Head, frontal view; **D**. Head, dorsal view; **E**. Pronotum, scutum and scutellum, dorsal view; **F**. Propodeum, posterodorsal view; **G**. Pygidial plate, dorsal view; **H**. Thorax, lateral view; **I**. Forewing; **J**. Hind leg; **K**. Male genitalia, ventral view; **L**. Male genitalia, dorsal view; **M**. Male genitalia, lateral view. Scale bars: 1.0 mm (**A–J**); 0.5 mm (**K–M**).

***Head***. Base of mandible deeply cut near base to give rise to two teeth-like prominences, distinctly incised at base on outer margin (Fig. 3B). Clypeus coriaceous; anterior margin of clypeus in middle distinctly, arcuately excavated, with sides of excavation acutely produced, with a small trapezi­form prominence mesally, and with subquadrate polished area on surface (Fig. 3B). Frons densely, finely punctate (PIS > PD), clearly divided into two parts by median frontal sulcus. Vertex densely punctate (PIS > PD), coriaceous (Fig. 3D). Inner orbits of eyes parallel at vertex. Antenna thin, F2 longer than scape.

***Mesosoma***. Pronotal collar lower than scutum, with three prominent tubercles, propleuron with oblique furrow containing conspicuous carinae. Scutum with dense punctures, PIS on average less than PD, anterior third of scutum with two medial parallel carinae diverging posteriorly; scutum with medial longitudinal depression; scutellum and metanotum with fine dense punctures, PIS on average more than PD (Fig. 3E). Mesopleuron convex, with dense shallow punctures (PIS > PD) (Fig. 3H). Propodeal dorsum with sinuate median carina not extending close to posterior margin, lateral to median carina with irregulate reticulate carinae (Fig. 3E), posterior surface with narrow wedge-shaped deep median furrow, sides of furrow with somewhat sinuate rugae (Fig. 3F); lateral surface of propodeum finely rugulose (Fig. 3H).

***Legs***. Hind tibia with 7–9 spines on posterior external margin (Fig. 3J).

***Wings***. Forewing with infumation crossing two submarginal cells and apex of the second discoidal cell; length relation between abscissae of radial vein of forewing: 2 < 5 < 3 < 1 ≤ 4 (Fig. 3I).

***Metasoma***. Basal platform of first segment approximately obconical with constriction, with conspicuous edges; gaster micropunctate with microscopic setae, surface alutaceous and shiny; pygidial plate short and not incised at apex, its lateral carina somewhat rounded; sternum VIII without median tooth at apex (Fig. 3G). Penis valve as in (Fig. 3K, L), and gonostyle as in (Fig. 3M).

**Female**. Unkown.

##### Distribution.

China (Yunnan).

##### Etymology.

The specific epithet *curvicarina* is derived from the Latin “*curv*-” (curved) and “*carina*” (carina), referring to the curved carinae on the propodeum of this species.

#### Lyroda quadratidens

Taxon classificationAnimaliaHymenopteraCrabronidae

Li & Li sp. nov.

39634BEE-F91B-5385-BE86-7DA00E1682D5

https://zoobank.org/F76F7F01-8AE7-4A9F-AA99-76A544667377

Fig. 4

##### Type material.

***Holotype*: China** • ♂; Guangxi, Hechi, Mulun National Nature Reserve; 25°08'N, 108°01'E; 18–19.VII.2015; coll. YiCheng Li; Yellow Pan Trap (YNAU). ***Paratypes***. • 10♂♂; same data as for holotype.

##### Diagnosis.

This new species is similar to *L. venusta* Bingham, 1897 but can be distinguished by the following characteristics: in the new species, lateral area of propodeum densely punctate and shagreened; posterior part of basal platform of the first gastral tergite with two longitudinal carinae on each side, scutellum densely covered with small punctures with smooth interstices, and the seventh gastral sternite without a median tooth-like process. To the contrary, in *L. venusta* lateral area of propodeum covered with fine, dense rugae-striae, posterior part of the basal platform with six longitudinal carinae on each side, scutellum sparsely covered with large punctures with microstriate interstices; and the seventh gastral sternite with a median tooth-like process.

##### Description.

**Male. *Measurements***. ♂, BL 9.2–11.1 mm. HW: HL: IODv: A3 = 50: 40: 26: 11; ODD: POD: Od: OOD = 7: 19: 8: 17.

***Color pattern***. Black; mandible brown, tibial spurs and tarsal claws more or less ferruginous; wings hyaline, slightly infumated at two external submarginal cells and at apex of second discoidal cell, veins testaceous, stigma, blackish brown. Setae silvery; dense on clypeus, base of mandible, antenna, gena, frons, vertex behind eye, tibiae, femora, posterior and posterolateral area of scutum, lateral margin of scutellum and metanotum, all sides of propodeum, and sternal areas of thorax; continuous silvery band of pile on Gt2–Gt3, setae extensively distributed on Gt1, more densely distributed at apex, forming band of pile; body extensively pilose, pygidium with dense, stiff brown setae mixed with longer erect brown bristles.

**Figure 4.**
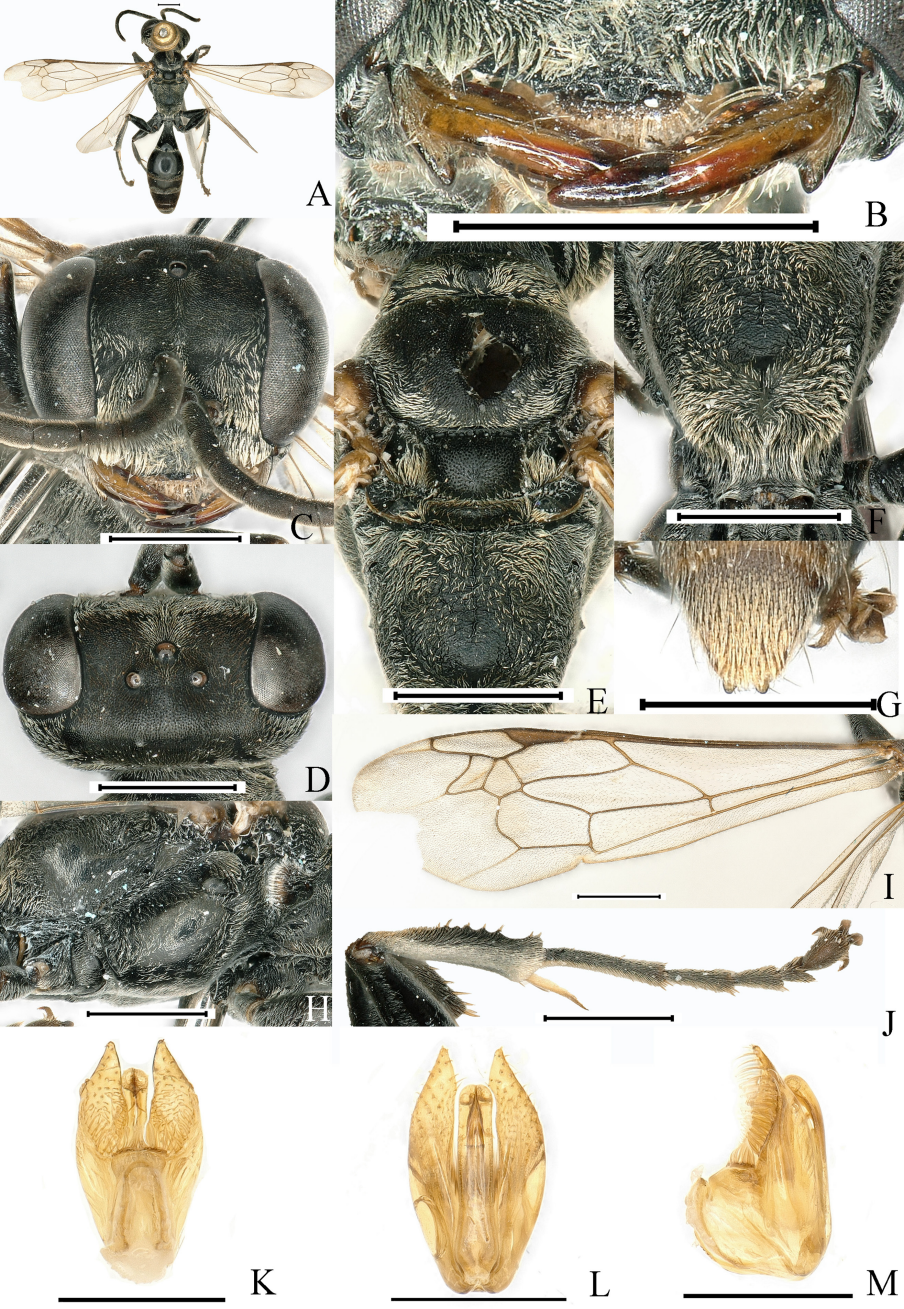
*Lyroda quadratidens* Li & Li, sp. nov. Male. Holotype. **A**. Habitus, dorsal view; **B**. Clypeus and mandible; **C**. Head, frontal view; **D**. Head, dorsal view; **E**. Pronotum, scutum and scutellum, dorsal view; **F**. Propodeum, posterodorsal view; **G**. Pygidial plate, dorsal view; **H**. Thorax, lateral view; **I**. Forewing; **J**. Hind leg; **K**. Male genitalia, ventral view; **L**. Male genitalia, dorsal view; **M**. Male genitalia, lateral view. Scale bars: 1.0 mm (**A–J**); 0.5 mm (**K–M**).

***Head***. Base of mandible deeply cut near base to give rise to two teeth-like prominences, conspicuously incised at base on outer margin (Fig. 4B). Clypeus coriaceous; anterior margin of clypeus in middle conspicuously, arcuately excavated, with sides of excavation acutely produced, with a small trapezi­form prominence mesally, and with subquadrate polished area on surface (Fig. 4C). Frons densely, finely punctate (PIS < PD), clearly divided into two parts by median frontal sulcus (Fig. 4C). Vertex densely punctate (PIS > PD) (Fig. 4D). Inner orbits of eyes parallel at vertex. Antenna thin, F2 longer than scape.

***Mesosoma***. Pronotal collar lower than scutum, with three prominent tubercles, propleuron with inconspicuous oblique furrow. Scutum with dense punctures, PIS on average less than PD, anterior third of scutum with two medial parallel carinae diverging posteriorly; scutum with medial longitudinal depression; scutellum and metanotum with fine dense punctures, PIS on average more than PD (Fig. 4E). Mesopleuron convex, with fine punctures (PIS > PD) (Fig. 4H); Propodeum. dorsum with sinuate median carina not extending close to posterior margin, lateral to median carina with irregulate reticulate rugae (Fig. 4E), posterior surface with narrow wedge- shaped deep median furrow, sides of furrow with somewhat sinuate rugae (Fig. 4F); lateral surface of propodeum densely, finely punctate (Fig. 4H).

***Legs***. Hind tibia with 7–9 spines on posterior external margin (Fig. 4J).

***Wings***. Forewing with infumation crossing two submarginal cells and apex of the second discoidal cell; length relation between abscissae of radial vein of forewing: 2 < 5 < 3 < 1 < 4 (Fig. 4I).

***Metasoma***. Basal platform of first segment approximately obconical with constriction, having conspicuous edges; gaster micropunctate with microscopic setae, surface alutaceous and shiny; pygidial plate with lateral carina; sternum VIII without median tooth at apex (Fig. 4F), penis valve as in (Fig. 4K, L), and gonostyle as in (Fig. 4M).

**Female**. Unknown.

##### Distribution.

China (Guangxi).

##### Etymology.

The specific epithet *quadratidens* is derived from the Latin “*quadrat*-” (square) and “*dens*” (toothed), referring to the square teeth on the anterior margin of the clypeus of this species.

## Supplementary Material

XML Treatment for Lyroda

XML Treatment for Lyroda multidentis

XML Treatment for Lyroda retirugosa

XML Treatment for Lyroda curvicarina

XML Treatment for Lyroda quadratidens

## Additional information

### Artificial Intelligence (AI) use

The authors accept full responsibility for the content of the manuscript, including the disclosure of any use of AI.

No AI tools were used in the preparation of this manuscript.

### Author ORCIDs

C. Li https://orcid.org/0009-0000-3149-1189

Q. Li https://orcid.org/0000-0001-5950-8843

L. Ma https://orcid.org/0000-0002-3436-1387

### Data availability

All of the data that support the findings of this study are available in the main text.
